# Efficacy of transdermal delivery of liposomal micronutrients through body oil massage on neurodevelopmental and micronutrient deficiency status in infants: results of a randomized placebo-controlled clinical trial

**DOI:** 10.1186/s40795-021-00458-8

**Published:** 2021-09-08

**Authors:** Aditi Apte, Mudra Kapoor, Sadanand Naik, Himangi Lubree, Pooja Khamkar, Diksha Singh, Dhiraj Agarwal, Sudipto Roy, Anand Kawade, Sanjay Juvekar, Rinti Banerjee, Ashish Bavdekar

**Affiliations:** 1 Department of Biosciences and Bioengineering, Nanomedicine Laboratory, Indian Institute of Technology Bombay, Powai, Mumbai, India; 2 PRERNA Young Scientist, KEM Hospital Research Centre, Pune, India; 3 KEM Hospital, Pune, India; 4 Vadu Rural Health Program, KEM Hospital Research Centre, Pune, India

**Keywords:** Anemia, Body massage, Fortification, Iron, Vitamin D

## Abstract

**Background:**

Micronutrient deficiency is a known cause of adverse neurodevelopment and growth. Poor adherence to oral regimes of micronutrient supplements is a known challenge during the implementation of supplementation programs. The present study evaluates the benefits of liposomal encapsulated micronutrient fortified body oils (LMF oil) that can be used for infant body massage in terms of neurodevelopment and prevention of deficiency.

**Study design:**

Double-blind randomized clinical trial.

**Methods:**

A total of 444 healthy infants aged 4-7 weeks were randomized to receive either LMF oil (containing iron, vitamin D, folate, and vitamin B12) or placebo oil for gentle body massage till 12 months of age. Blood samples were collected at 6 and 12 months for transferrin saturation (TSAT), hemoglobin, and 25-hydroxy vitamin (25-OH-D) levels. Mental and motor development was assessed at 12 months using developmental assessment for Indian Infants (DASII).

**Results:**

A total of 391 infants completed the study. There was no significant improvement in the hemoglobin in the intervention group at 12 months of age as compared to the placebo group [− 0.50 vs.-0.54 g%]. There was a marginally significant improvement in 25-OH-D at 12 months in the LMF oil group [+ 1.46vs.-0.18 ng/ml, *p* = 0.049]. In the subgroup of infants with moderate anemia, the intervention prevented the decline in hemoglobin at 12 months of age [adjusted mean change + 0.11vs.-0.51 g%, *p* = 0.043]. The mental or motor developmental quotients in the intervention group were not significantly different from those in the placebo group.

**Conclusion:**

Use of LMF oil for prevention of nutritional deficiency did not offer significant protection against nutritional anemia but prevented vitamin D deficiency to some extent with improvement in 25-OH-D at 12 months. In the subgroup of infants with moderate anemia, the intervention prevented the decline in hemoglobin at 12 months of age. The intervention did not result in significant improvement in mental or motor development. Further evaluation with increased doses needs to be undertaken.

**Trial registration:**

CTRI no: CTRI/2017/11/010710; dated 30/11/2017.

**Supplementary Information:**

The online version contains supplementary material available at 10.1186/s40795-021-00458-8.

## Introduction

Early childhood is a critical period for growth and neurodevelopment [1]. There is compelling evidence that iron deficiency during 6–24 months of age is associated with poor cognitive, motor, and socioemotional development [2]. Folate, known to have a role in neural tube development, and vitamin B12, known to affect myelination and methionine synthesis, are important micronutrients for brain development [3–5]. There is upcoming evidence on the benefits of vitamin D in neurodevelopment apart from its effect on bone and calcium metabolism [6]. Despite this, there is inconsistent evidence from randomized controlled studies on whether supplementation of one or more of these nutrients can result in improved neurodevelopment [7, 8].

Potential for improved neurodevelopment is one of the important rationales behind the introduction of iron-folate supplementation in public health programs and despite the presence of these programs, 59% of under-five children in India suffer from nutritional anemia [9]. Vitamin D deficiency has been documented in up to 80% of the Indian population of all ages [10]. Poor adherence to dosage regimes due to forgetfulness of the mothers, fear of side effects like gastritis, especially for iron supplements, poor coverage, and lack of knowledge are known challenges in the implementation of supplementation programs [11–13]. A small amount of orally administered iron is absorbed, and the unabsorbed iron is associated with adverse effects such as gastritis and increased risk of gastrointestinal infections [14, 15].

Transdermal route is an alternate non-invasive route for the administration of nutrients and drugs with gastrointestinal intolerability and offers higher surface area and may offer better compliance as compared to the oral route. Although there are a few molecules that can favorably cross the intact skin barrier, active and passive technologies can potentially improve transdermal absorption of several molecules [16–20]. We developed an innovative formulation of body oil containing soya phosphatidylcholine and oleic acid-based liposomes encapsulating iron, folate, vitamin B12, and vitamin D using a patented platform for increased permeation across the stratum corneum barrier [21, 22]. The liposomes showed high permeation through the stratum corneum and showed 40–50% encapsulation efficiency for ferrous bisglycinate, 20–40% for folate and B12, and 90–95% for vitamin D. The technology has demonstrated safety and efficacy in in-vitro and in-vivo animal models for transdermal delivery of nutrients at supplemental doses [23]. The technology was also found to be non-irritant in healthy human volunteers and children [24, 25].

This technology was used to fortify body oil to be used for infant massages. Infant oil massage is an ancient practice in the Indian subcontinent with some benefits for growth and development and has widespread cultural as well as social acceptance [26]. A formative research study conducted before the clinical study within the study area showed that infant oil massage was a routine practice in more than 90% of households and there was a readiness to accept fortified oil [23]. Thus fortifying the body oil with nutrients can potentially improve the compliance and acceptability of the intervention.

This paper describes a proof of concept evaluation of this liposomal encapsulated micronutrient fortified oil (LMF oil) in healthy infants from rural India. The objectives of the study were to assess whether the use of LMF oil for gentle body massage during infancy results in improved neurodevelopment with reduced risk of nutritional deficiency at the end of 1 year.

## Methods

### Study design and settings

This was a randomized, double-blind, placebo-controlled parallel-group clinical study that was conducted between February 2018 to September 2019 at Vadu Rural Health Program of Kind Edward Memorial (KEM) Hospital Research Centre, Pune, India [24].

### Approvals

The study was approved by the institutional ethics committee of KEM Hospital Research Centre (KEMHRC/LFG/EC/146) and the Health Ministry Screening Committee of India. The study was registered at the Clinical Trial Registry of India (CTRI no: CTRI/2017/11/010710, dated 30/11/2017 accessed at http://ctri.nic.in/Clinicaltrials/showallp.php?mid1=21315&EncHid=&userName=oil).

### Study population

Healthy infants born at full term aged between 4 to 7 weeks with a birth weight of at least 2 kg were approached for recruitment in the study from the study population at Vadu Rural Health Program (VRHP). VRHP is a community-based program for research and health care delivery by KEM Hospital Research Centre, Pune, India, and is functional across 22 villages in Western Maharashtra. The population is mainly rural with agriculture as their main occupation. Demographics of this population are regularly monitored through health and demographic surveillance (HDSS) conducted by VRHP [27]. Based on the HDSS data, antenatal care data from the public and private health facilities and the number of births within Vadu HDSS area were tracked and potential study participants were enumerated. Written informed consent was taken from the parents of eligible infants and those willing to participate were enrolled in the study. Major exclusion criteria included generalized skin disorder or skin infection, major congenital anomalies, major birth or neonatal complications, and severe malnourishment (weight for age Z-score < − 3.0). Families who were not planning to relocate for at least 1 year were included in the study.

### Preparation of the used oil

The formulation of LMF oil was developed initially by Banerjee et al. at the Indian Institute of Technology, Mumbai, India. The technology was licensed to KEM Hospital Research Centre for non-commercial use and Murli Krishna Pharmaceuticals Ltd. for manufacturing and commercialization. The LMF oil contained sunflower oil fortified with liposomal encapsulated ferrous bisglycinate, vitamin B12, folic acid, and vitamin D. Sunflower oil was used as a base as it is a rich source of essential fatty acids like linoleic acid [28]. The micronutrients were added as per their recommended dietary allowance for the given age [29, 30].

Ferrous bisglycinate was procured from Puneet Laboratories, Mumbai, India and vitamin B12(cyanocobalamin), folate, and vitamin D(1,25-dihydroxy cholecalciferol) were procured from SRL Chemicals Pvt. Ltd., Mumbai, India. Soya lecithin and Oleic acid were procured from Hi-Media. Sunflower oil was obtained from Prasukh Udyog Pvt. Ltd., Pune which provided sunflower oil extracted without use of any chemical process or refining. The liposomes of the nutrients were prepared using generally recognized as safe (GRAS) materials without using any organic solvents. The liposomal oils were manufactured by Murli Krishna Pharma Pvt. Ltd., Ranjangaon, Pune under EuGMP conditions.

Two strengths of the test oil were developed for age groups 1–6 months and 6–12 months respectively to match their dietary requirements of individual nutrients (Additional Table 1). The technology was earlier tested for its safety and efficacy through in-vitro and in-vivo studies for transdermal delivery of supplemental doses of nutrients [23] (Additional Table 2). The process of development of the LMF oil as a study intervention has been described earlier [24].

The placebo oil was an identical-looking product containing sunflower oil which was fortified with empty liposomes (not containing micronutrients) and was identical in appearance and smell to that of the test oil. Both the formulations were stable at room temperature when kept away from sunlight for at least 3 months.

### Study procedures

After enrollment, the infants were randomized in a 1:1 ratio to receive either LMF oil or placebo oil using computer-generated block randomization. To minimize the selection bias, allocation concealment was done by using sequentially numbered opaque envelopes.

The caregivers from both groups were trained for gentle massage of a fixed amount of the study oil (LMF oil or placebo oil, 2.5 ml) on the skin avoiding contact with mucosal surfaces, face, neck, and scalp. The volume of oil application was decided based on a pilot study conducted to assess the adequate amount of sunflower oil for infants of various ages [24]. The caregivers were informed regarding the health benefits of gentle oil massage in infants to ensure good compliance to intervention. They were instructed to use the study oil at night before bedtime to allow optimum absorption of the study oil. Monthly supplies of oil bottles were provided through home visits by the field research assistants and compliance was monitored through compliance cards and calculation of used oil bottles.

Data collection was done at baseline, 6 and 12 months through clinic visits. At baseline, demographic details, birth history, maternal socioeconomic status were collected. Anthropometric parameters (weight, length, and head circumference) were measured using standardized procedures. Information about breastfeeding and weaning was collected at each clinic visit. Details about any concomitant medicines including iron or vitamin supplements and adverse events were collected during routine fortnightly field visits by the field research assistants.

All the study participants underwent neurodevelopmental assessment using a developmental assessment scale for Indian infants (DASII) at 12 months by a trained clinical psychologist. This scale is an Indian modification of the Bayley scale for infant development containing mental (163 items) and motor (67 items) scales and can be used to assess mental and motor developmental quotient in infants aged up to 30 months [31]. The assessor was blinded to the allocation of groups.

Tolerability of the study oils was assessed using the standard skin scoring system for irritation which grades the presence of erythema and dryness during clinic visits [32]. All the caregivers were given appropriate nutritional advice during clinic visits and children with unresolved anemia or vitamin D deficiency were treated with oral iron and vitamin D supplements at the end of the study.

### Laboratory procedures

At 6 and 12 months, 5 ml of non-fasting venous blood samples were collected into evacuated tubes containing EDTA and trace element-free tubes containing no coagulant (BD Vacutainer). Blood in the EDTA tubes was immediately used for assessment of hemoglobin (Hb) concentrations and red blood cell on cell analyzer at the research laboratory at VRHP with appropriate quality control and standard procedure. The trace element-free tubes were centrifuged at 3000 rpm for 15 mins at 4 °C, and serum was separated, aliquoted, and stored at -80 °C till further analysis. The serum samples were transported in cooling boxes with ice packs to the biochemistry laboratory at KEM Hospital, Pune in batches for assessment of transferrin saturation (TSAT) and 25-hydroxy vitamin (25-OHD) levels.

Complete blood counts were assessed using a duly calibrated Medionic M3 auto-analyzer. Iron and total iron-binding capacity (TIBC) estimations were done using the Ferrozine method on Cobas-Integra 400-plus analyzer [33]. The inter-and intra-batch coefficient of variations for Iron were 3.4 and 3.0% respectively and for TIBC were 5.7 and 5.1% respectively. The TSAT levels were calculated as serum iron divided by TIBC.

Serum 25-OHD estimation was done using chemiluminescent microparticle immunoassay technique on Architect ci4000 Chemistry analyzer (Abbott) [34]. The inter- and intra-batch coefficients of variations for vitamin D were 7.2 and 6.6% respectively.

### Data management and quality control

The data were collected on paper case record forms and were then entered in electronic case record forms using Open Clinica community edition software after source data verification. The data entered into the electronic case record form (eCRF) were checked for completeness and quality assurance. The anonymized dataset was extracted for analysis following source data verification. The dataset was archived at a local server at VRHP.

### Sample size calculation and data analysis

The primary outcome for the study was a neurodevelopmental assessment using DASII. Hence, sample size calculation was based on the assumption that the intervention would improve the developmental quotient on DASII by at least a score of 5. Considering a standard deviation of 15 for DASII score, alpha error of 5%, and power of 90, sample size of 189 was required in each group. Considering a dropout rate of 15%, the sample size of 222 in each arm was selected. This sample size was adequate to detect a 27% reduction in the prevalence of anemia in the test group considering 60% prevalence of anemia in the placebo group and 20% reduction in the prevalence of vitamin D deficiency considering vitamin D deficiency (< 20 ng/ml) in 75% of the participants in the placebo group at 90% power and 5% alpha error.

The data analysis was done on an intention-to-treat basis. Parameters were expressed as the mean and standard deviation for normally distributed variables and median with interquartile range for non-parametrically distributed variables. Normally distributed variables were compared using an unpaired t-test with unequal variance. For data not normally distributed even after square root transformation, non-parametric tests were used. Proportions were compared between the groups by using the Chi-square test.

Linear regression models were used for calculating treatment effect in terms of the mental and motor developmental quotient, change in hemoglobin, change in 25-hydroxy vitamin D levels, and change in transferrin saturation levels between the two groups while adjusting for compliance and concomitant medications. Additionally, multiple logistic regression analysis was done to compare the effect of the intervention on subgroups of motor and mental developmental quotient (DQ) basing the categories on the 50^th^ centile. In addition to adjustment for compliance to intervention and concomitant nutritional supplements, the subgroup analyses were adjusted for gender, weaning age, and maternal education. All the analyses were done using STATA version 15.0.

## Results

**Fig. 1:**
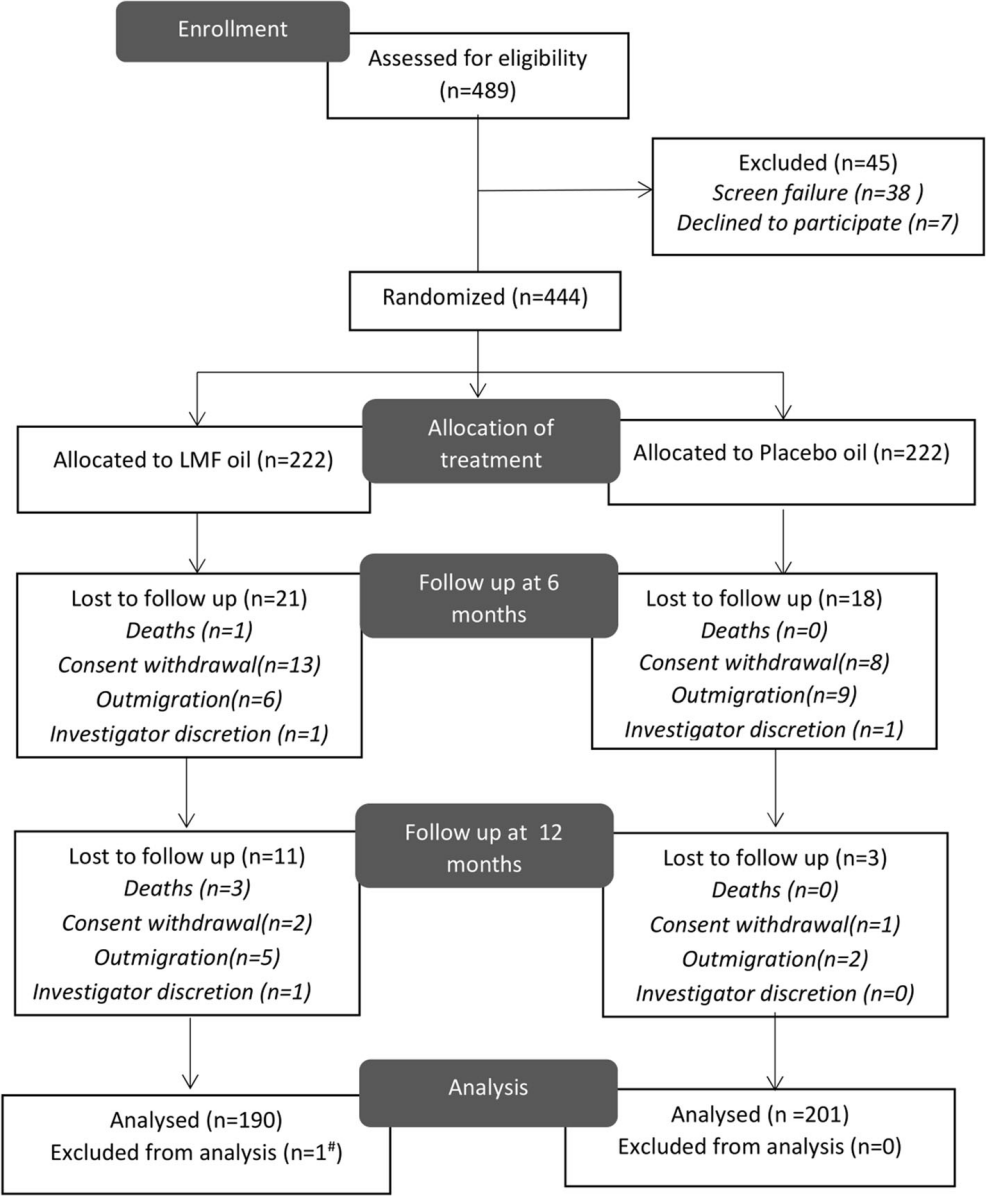
Study flow chart. ^#^ insufficient blood sample, LMF oil – Liposomal encapsulated micronutrient fortified oil

Of the total 489 children aged 4–7 weeks of age screened at baseline, 45 children failed to meet eligibility criteria. Total 444 children were randomly assigned to the study groups. At 6 months, 405 children completed clinic visit with attrition rate of 9.45% (*n* = 21) in test and 8.11% (*n* = 18) in placebo group (*p* = 0.62). At 12 months, a total of 391 children completed the study with an overall attrition rate of 14.4% in the test group and 9.45% in the placebo group (*p* = 0.94). The reasons for loss to follow-up mainly included outmigration and consent withdrawals (Fig. 1).

Baseline demographic information including maternal characteristics is given in Table 1. All the demographic parameters and birth, as well as maternal characteristics, were comparable between the groups except that the body weight was more in the LMF oil group. About 66% of the children were born vaginally and 13–17% of the children were born with low birth weight (< 2.5 kg). The average maternal age was approximately 24 yrs. More than 95% of the mothers received some school education and approximately 94% of them were homemakers.

**Table 1:** Baseline demographic information of the study participants^a^

	LMF oil (*n* = 222)	Placebo oil (***n*** = 222)
**Age in days**^**$**^	35.59 ± 5.86	37.51 ± 6.14
**Sex, number of females**	97(43.69)	107(48.20)
**Weight in kg**^b^	3.97 ± 0.52	3.88 ± 0.49
**Length in cm**	54.60 ± 2.15	54.47 ± 2.37
**Head circumference in cm**^**$**^	35.95 ± 1.48	35.88 ± 1.46
**Children with low birth weight**^c^	29(13.06)	37(16.66)
**Mode of delivery**		
*Vaginal*	146(65.77)	146(65.77)
*Caesarean section*	76(34.23)	76(34.23)
**Exclusively breastfed**	212(95.50)	215(96.85)
**Birth order**		
*First*	65(29.28)	73(32.43)
*Second*	88(39.64)	87(39.19)
*Third*	45(20.27)	49(22.07)
*Fourth*	20(09.01)	9(4.05)
*Fifth or more*	4(1.80)	5(2.25)
**Maternal age in years**^**$**^	24.30 ± 3.79	24.34 ± 3.79
**Maternal education**		
*Illiterate*	4(1.8)	9(4.05)
*Primary education*	46(20.72)	52(23.42)
*Secondary education*	59(26.58)	50(22.52)
*Higher secondary education*	76(34.23)	58(26.13)
*Graduate*	31(13.96)	48(21.17)
*Postgraduate*	6(2.7)	6(2.7)
**Maternal occupation**		
*Homemaker*	208(93.69)	208(93.24)
*Agriculture*	5(2.25)	6(2.70)
*Student*	–	1(0.45)
*Industry*	–	3(1.35)
*Others*	9(4.05)	5(2.25)

Unpaired t-test used for comparison of normally distributed variables; Pearson chi2 for frequencies; ^a^ Values expressed as mean+/−SD for continuous data and n(%) for categorical data; ^b^*p* = 0.03; $Variables not normally distributed; Kolmogorov Smirnoff test used for comparison; ^c^- birth weight < 2.5 kg, *LMF oil* Liposomal encapsulated micronutrient fortified oil

More than 90% of the participants in both the groups had compliance to the intervention of more than 80% (Additional Figure 1). Compliance with the intervention was comparable between the groups at 6 months and 12 months.

Based on the data about concomitant medications received during study duration, 14(7.36%) and 9(4.47%) children from the LMF oil and placebo oil group respectively received health supplements containing iron for at least 1 month during the study period. On the other hand, 103 (54.21%) and 112 (55.72%) children in the LMF oil and placebo oil group respectively received additional vitamin D supplementation for at least 1 month. These values were not significantly different between the two groups.

**Fig. 2:**
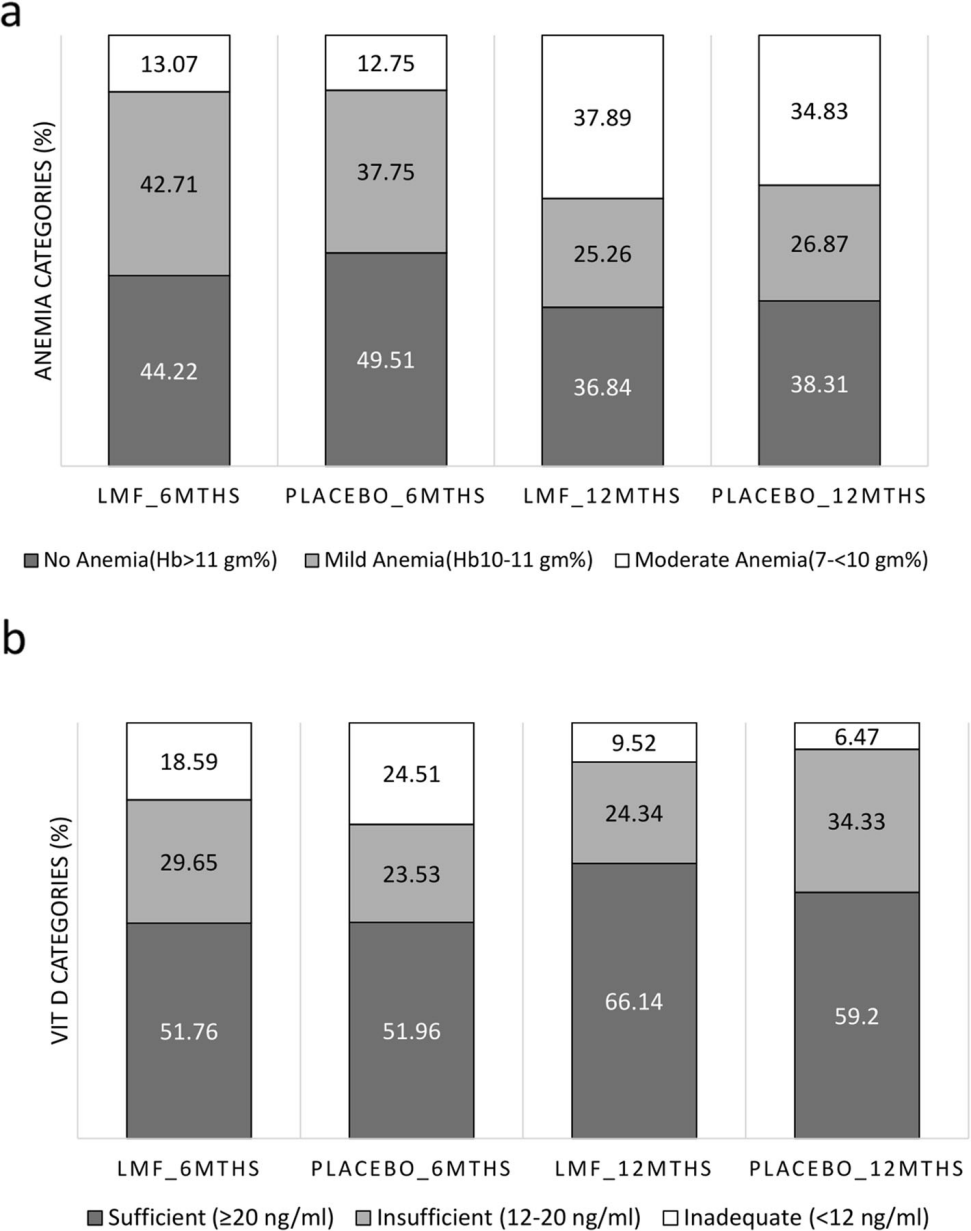
Distribution of (**a**) anemia and (**b**) vitamin D deficiency at 6 months and 12 months in LMF oil and placebo oil groups. LMF oil – Liposomal encapsulated micronutrient fortified oil; Classification of anemia: Mild (Hb 10–11 g%), Moderate (Hb 7- < 10 g%), Severe (Hb < 7 g%)]; 25-Hydroxy vitamin D levels: Sufficient (≥20 ng/ml); Insufficient (12–20 ng/ml); Inadequate (< 12 ng/ml)

**Fig. 3:**
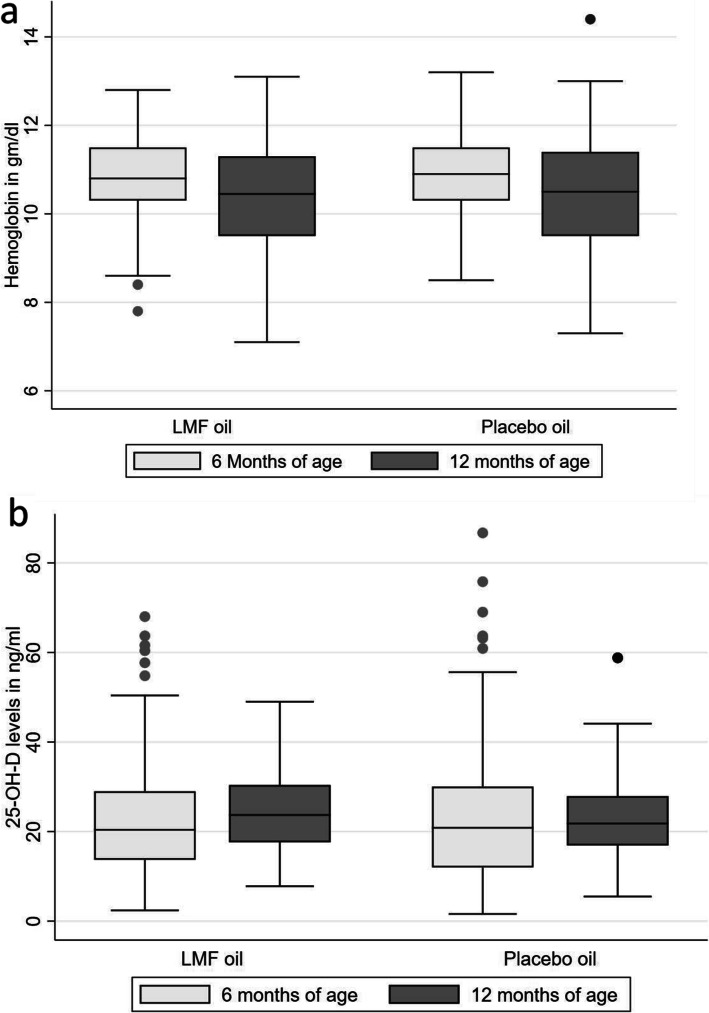
Change in (**a**) hemoglobin and (**b**) 25-hydroxy vitamin D levels in LMF oil and placebo oil group. LMF oil – Liposomal encapsulated micronutrient fortified oil; The horizontal line in the middle of the box is the median value and the lower and upper boundaries indicate the 25^﻿th^ and 75^﻿th^ percentiles, respectively. Values more than 1.5 box-lengths from the lower edge of the box are considered as outliers and are designated with circles. The lines (whiskers) drawn at the ends of each plot indicate the largest and smallest observed values that are not outliers

There was a mean hemoglobin reduction of 0.5 g% in the LMF oil group at 12 months which was comparable to that in the placebo oil group (0.54 g%) (Table 2 & Fig. 3a). Overall, the proportion of anemia (Hb < 11 g/dl) increased at 12 months as compared to 6 months without any significant difference between the study groups (Table 2). As compared to 6 months, there was a 7.67 and 11.2% increase in anemia in LMF oil and placebo oil groups respectively at 12 months of age (Fig. 2a). In the subgroup of children with moderate anemia at 6 months, the intervention of LMF oil prevented the reduction in hemoglobin at 12 months as compared to the placebo oil group [0.11 g% increase in LMF oil group vs. 0.51 g% reduction in the placebo oil group, *p* = 0.043] (Table 2). None of the children had severe anemia at 6 or 12 months. There was a reduction in TSAT levels in both the groups at 12 months as compared to 6 months without any significant intergroup difference [− 2.36% in LMF oil group Vs. -2.10 in placebo oil group].

**Table 2:** Changes in the hematological and biochemical parameters^a^

	LMF oil			Placebo oil			Treatment effect (95% CI)
	N	Value	Adjusted mean change^b^	N	Value	Adjusted mean change^b^	
**Changes in hemoglobin (Hb) levels, gm%**							
Hb at 6 months	199	10.84 ± 0.88	−0.50	204	10.96 ± 0.90	− 0.54	− 0.01(− 0.26–0.28)
Hb at 12 months	190	10.36 ± 1.26		201	10.42 ± 1.30		
*Subgroup of children with no anemia* (Hb > 11 g%) *at 6 months*^e^							
Hb at 6 months	88	11.61 ± 0.41	−0.70	101	11.68 ± 0.55	− 0.62	−0.08(− 0.38–0.23)
Hb at 12 months	84	10.90 ± 1.03		99	11.05 ± 1.09		
*Subgroup of children with mild anemia* (Hb 10–11 g%) *at 6 months*^e^							
Hb at 6 months	85	10.51 ± 0.28	−0.44	77	10.52 ± 0.32	− 0.41	−0.03(− 0.37–0.31)
Hb at 12 months	84	10.08 ± 1.23		76	10.08 ± 1.10		
*Subgroup of children with moderate anemia*(Hb 7- < 10 g%) *at 6 months*^e^							
Hb at 6 months	26	9.31 ± 0.56	0.11	26	9.49 ± 0.33	− 0.51	0.62(0.02–1.22)^c^
Hb at 12 months	22	9.34 ± 1.26		26	8.98 ± 1.04		
**Changes in transferrin saturation (TSAT) levels, %**							
TSAT at 6 months	199	12.1(7.89–15.9)	−2.36	204	10.9(7.9–15.45)	−2.10	−0.26(−1.89–1.36)
TSAT at 12 months	189	7.9(5.9–13.3)		201	8.1(5.8–11.7)		
**Change in 25-Hydroxy vitamin D (25-OH-D) levels, ng/ml**							
25 OH D at 6 months	199	20.4(13.7–29)	1.46	204	20.85(12–30.05)	−0.18	1.63(− 0.52–3.78)^d^
25OH D at 12 months	189	23.7(17.6–30.4)		201	21.80(16.9–27.9)		

^a^Values expressed as Mean+/−SD for parametric data and median (interquartile range) for nonparametric data; ^b^Parameters adjusted for compliance to the intervention, concomitant administration of iron and vitamin D supplements; ^c^*p* = 0.043, ^d^ = 0.049; ^e^-Subgroup analysis additionally adjusted for gender, weaning age and maternal education, *LMF oil* Liposomal encapsulated micronutrient fortified oil

The intervention of LMF oil resulted in significantly increased 25-OHD levels at 12 months in the LMF oil group as compared to a decrease in 25-OHD levels in the placebo oil group after adjusting for intake of concomitant oral vitamin D supplements [1.46 ng/ml vs. -0.18 ng/ml, *p* = 0.049] (Table 2 & Fig. 3b). At 12 months, the proportion of children with vitamin D insufficiency (25-OHD levels < 20 ng/ml) reduced by 14.38% in the intervention group as compared to a 7.24% decrease in the placebo oil group (*p* = 0.07) (Fig. 2b).

**Fig. 4:**
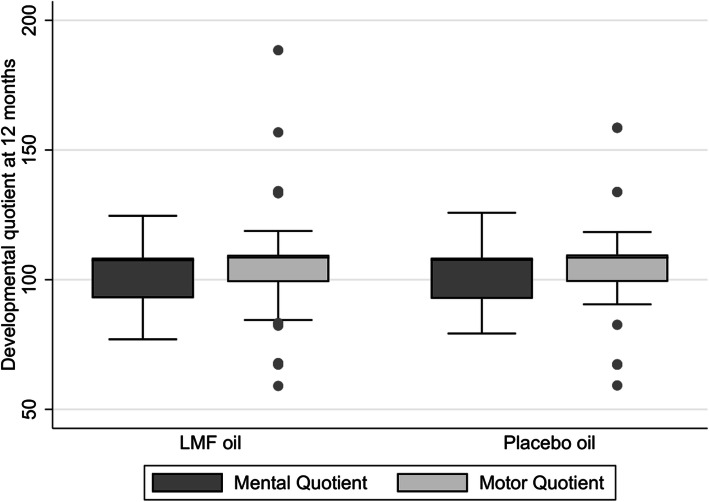
Developmental assessment at 12 months using developmental assessment of Indian Infants (DASII) scale. LMF oil – Liposomal encapsulated micronutrient fortified oil; The horizontal line in the middle of the box is the median value and the lower and upper boundaries indicate the 25^﻿th^ and 75^﻿th^ percentiles, respectively. Values more than 1.5 box-lengths from the lower edge of the box are considered as outliers and are designated with circles. The lines (whiskers) drawn at the ends of each plot indicate the largest and smallest observed values that are not outliers

There was no significant difference in the mental DQ between the groups at 12 months of age [Median (IQR); 107.49(92.93–108.38) in the LMF oil group vs. 107.57(99.66–108.38) in placebo oil group; *p* = 0.56]. Similarly, motor DQs were comparable between the groups at 12 months of age [Median (IQR); 108.56(99.09–109.57) in the LMF oil group vs. 108.49(99.25–109.66) in placebo oil group; *p* = 0.58] (Fig. 4).

During subgroup analysis for individual clusters of motor development, children in the LMF oil group were rated significantly better for the social interaction cluster as compared to the placebo oil group. In the subgroup of children with vitamin D insufficiency, the motor developmental quotient was significantly higher in the LMF oil group as compared to placebo oil (Table 3). There were no significant differences for mental developmental quotient in the subgroup analysis [Data not shown].

**Table 3:** Odds ratios for more than median (50^﻿th^ centile) developmental score

Parameter	N	OR for placebo oil	OR for LMF oil	95% CI
Motor DQ^a^	391	1	1.09	0.73–1.64
Mental DQ^a^	391	1	0.97	0.65–1.44
**Subgroup analysis for motor DQ**^b^				
Hemoglobin at 6 months				
*No anemia*	182	1	1.04	0.56–1.95
*Mild anemia*	155	1	1.72	0.85–3.50
*Moderate anemia*	48	1	0.64	0.15–2.80
Transferrin saturation at 6 months				
*TSAT < 16%*	299	1	1.09	0.68–1.76
*TSAT > 16%*	88	1	1.10	0.43–2.83
Vitamin D levels at 6 months				
*Sufficient (≥20 ng/ml)*	202	1	0.83	0.46–1.49
*Insufficient (12–20 ng/ml)*	102	1	3.38	1.30–8.82*
*Inadequate (< 12 ng/ml)*	83	1	0.66	0.24–1.82
**Analysis for individual clusters of DASII**^a^				
Social interaction	390	1	1.51	1.00–2.29 **
Locomotion: Coordinated movements	390	1	1.02	0.66–1.58
Language and vocabulary	390	1	1.25	0.81–1.94
Body control	390	1	1.55	0.73–3.28

* *p* = 0.013, ** *p* = 0.047;^a^ Multiple logistic regression adjusted for concomitant medicines and compliance; ^b^ For subgroups, analysis additionally adjusted for gender, weaning age, and maternal education; *LMF oil* Liposomal encapsulated micronutrient fortified oil, *DQ* Developmental quotient, *DASII* Developmental assessment of Indian infants, *TSAT* Transferrin saturation, *OR* Odd’s ratio

The anthropometric parameters were comparable between the groups at all timepoints except that the head circumference of children in the LMF oil group was significantly greater than the placebo oil group at baseline. The average age of weaning was approximately 6 months in both the groups (Additional Table 3).

The local and systemic adverse events were not significantly different between the groups (Additional Table 4).

## Discussion

This was the first proof-of-concept study to assess the benefit of liposomal micronutrient fortified oil in the prevention of nutritional deficiencies and improvement in neurodevelopment.

Our study showed that the use of LMF oil for infant massage did not significantly prevent nutritional anemia, however, showed some protection against anemia in children with moderate anemia at 6 months of age. The composition of LMF oil was based on the recommended daily amounts of absorbed elemental iron and other nutrients for the given age and based on available in vitro as well as in vivo evidence. Higher than recommended daily amounts were not used to avoid the risk of toxicity. Further, absorption of iron is dependent upon the iron stores in the body, thus the absorption from the oral route is increased in deficiency states [35]. Although, there is no clear evidence about the regulation of the mechanism of absorption of iron through the transdermal route, this may probably explain the improvement in hemoglobin levels in the moderate anemia subgroup. To our knowledge, this is the first clinical study evaluating transdermal delivery of iron. Transdermal delivery of iron in the form of ferric pyrophosphate has been attempted earlier by Modepalli et al. in rat models using iontophoresis, microporation and electrophoresis techniques [36, 37]. However, there are no published clinical studies to evaluate safety and efficacy of these techniques. Transdermal patches for ferrous bisglycinate are available in the United States (Patch MD™) but lack any published safety and efficacy data [38].

There was a significant improvement in 25-OHD levels in the LMF oil group as compared to the placebo oil group. Vitamin D is fat-soluble and the lipophilic nature of the molecule facilitates liposomal encapsulation and transcutaneous absorption [39]. There have been preclinical and clinical studies in adults on topical formulations for vitamin D which have demonstrated the potential in transdermal delivery of vitamin D. This includes ex vivo studies done in animal and human cadaveric skin using topical formulations for vitamin D [40]. Few adult clinical studies have demonstrated benefit of topical use of vitamin D gel containing 5000 IU of cholecalciferol including a study by Sadat et al. in 50 female medical students [41] and a randomized clinical study done by Bubshait et al. [42]. TransEpi is a transdermal technology that has demonstrated efficacy in percutaneous delivery of vitamin D in a human pharmacokinetic study [43]. However, the doses used in the clinical studies were much higher than the recommended dietary intake.

The intervention of LMF oil did not result in significant improvement in the overall mental and motor developmental quotient. There is equivocal literature about the effect of iron supplements on cognitive and motor development in children under 2–3 years [2] and more long-term studies are needed in this regard. Nutritional interventions such as zinc, vitamin A, folate, or multiple micronutrients have been shown to have a small effect on mental development [8, 44]. In the present study, as the intervention did not result in significant improvement in nutritional anemia, the effect of the intervention on neurodevelopment through the reduction in anemia cannot be interpreted.

There was an improvement in the motor quotient after the intervention within the subgroup of insufficient vitamin D. Observational studies have shown an inconsistent association of 25-OHD with neuro-behavioral outcomes. There is a lack of evidence from randomized controlled studies that address this issue. Recent findings from a small study in 55 infants have shown a beneficial effect of oral vitamin D3 400 IU on gross motor development. However, this effect was not found to be dose-dependent as only a low dose was associated with the benefit [45]. It would be important to assess whether higher doses of vitamin D would provide significant benefits in vitamin D deficient children.

The study showed significant improvement in social interaction in the LMF oil group. Studies in preschool children have shown that iron deficiency anemia is associated with altered affect and behaviour [46] as well as poor externalizing behavior [47]. However, as the intervention of LMF oil did not significantly improve hemoglobin levels in the overall population, whether this effect was independent of improvement in anemia status remains to be understood.

The use of LMF oil was not found to be associated with increased gastrointestinal adverse effects as compared to placebo oil, unlike oral iron [48]. There was no significant increase in the rashes or pigmentation or skin infection with almost year-long use of LMF oil. However, there was a non-significant increase in the risk of respiratory infections with the use of LMF oil (Additional Table 2).

One of the strengths of the study is that it used a traditional age-old custom of an infant oil massage to deliver the intervention of micronutrients to babies and demonstrated very good compliance to the intervention (around 96%). This could be due to the social acceptability of infant oil massage in Indian rural communities. The study evaluated a technology paired with social innovation in a public health setup. The innovation has used only biodegradable GRAS-approved materials thus improving its acceptability and tolerability while use in infants. The cost of manufacturing LMF oil was minimal (approx. 1 $ for 90 ml) and the oils were stable at room temperature making it suitable for large-scale public use.

The study has several limitations. Although it was feasible to implement infant massage for 12 months in our study, in routine practice, infant massage is not regularly implemented in older infants and toddlers [49]. .It may also be challenging to administer oil massage to older infants due to their increased movement. Further, traditionally oil massage is administered to the babies in the morning before bath. It was hypothesized that this would not have allowed adequate time for the absorption of nutrients from the LMF oil. This limitation was overcome by requesting the caregivers to administer the oil at bedtime.

The nutrition status of breastfeeding infants is dependent on the maternal intake of nutrients which was not collected during the study. Vitamin D levels are affected by the duration and intensity of sun exposure which was not collected during the study. Ferritin and soluble transferrin receptor levels which are better indicators of iron stores and iron deficiency than hemoglobin and transferrin were not measured due to logistic constraints and the analysis for anemia were not adjusted for inflammatory markers like C-reactive proteins. Vitamin B12 and folate may have played a role in the developmental outcomes. However, this study has not measured changes in the plasma levels of folate and B12. Despite the lack of overall efficacy, this is the first scientific attempt in assessing iron delivery through the skin in a clinical study. Results of the study show that vitamin D due to its lipophilic nature is more suitable for transdermal delivery than iron. The transdermal delivery of iron needs to be studied using more mechanistic human studies to assess cutaneous pharmacokinetics of the liposomal encapsulated iron and in target populations with iron deficiency who are probably more iron-responsive than the healthy population. Also, the effect of increasing doses of nutrients needs to be studied for transdermal delivery.

## Conclusion

The intervention of liposomal encapsulated micronutrient fortified oil for supplementing micronutrients to infants through body massage did not prevent anemia or improve neurodevelopment in Indian infants but prevented vitamin D deficiency to some extent with improvement in 25-OHD at 12 months. In the subgroup of infants with moderate anemia, intervention prevented the decline in hemoglobin at 12 months of age. Future studies are needed to optimize the composition of oil and to assess the effect of increased doses.

## Supplementary Information

**Additional file 1: Table 1.** Micronutrient composition of LMF oil. **Table 2.** Tolerability and acceptability of LMF oil in adults and children. **Table 3.** Anthropometry and Nutritional Parameters at 6 months and 12 months. **Table 4.** Analysis of adverse events. **Figure 1.** Compliance to the intervention amongst LMF oil and placebo oil group.

## Data Availability

The datasets used and/or analyzed during the current study are available from the corresponding author on reasonable request.

## Abbreviations

LMF oil: Liposomal micronutrient fortified oil
KEM Hospital: King Edward Memorial Hospital
SRL: Super Religare Laboratories
GRAS: Generally recognized as safe
EuGMP: European Good Manufacturing Conditions
DASII: Developmental assessment scale for Indian Infants
EDTA: Ethylenediaminetetraacetic acid
TSAT: Transferrin saturation levels
25-OHD: 25-Hydroxy vitamin D
TIBC: Total iron binding capacity
eCRF: Electronic case record form
Hb: Hemoglobin
DQ: Developmental quotient
