# Supplementary material for: Efficacy of transdermal delivery of liposomal micronutrients through body oil massage on neurodevelopmental and micronutrient deficiency status in infants: results of a randomized placebo-controlled clinical trial
Source: BMC Nutr. 2021 Sep 8;7:48. doi: 10.1186/s40795-021-00458-8 (PMC8524365; doi:10.1186/s40795-021-00458-8)
Supplement: Supplementary file 1 — Additional file 1: Table 1. Micronutrient composition of LMF oil. Table 2. Tolerability and acceptability of LMF oil in adults and children. Table 3. Anthropometry and Nutritional Parameters at 6 months and 12 months. Table 4. Analysis of adverse events. Figure 1. Compliance to the intervention amongst LMF oil and placebo oil group. [file 40795_2021_458_MOESM1_ESM.docx]

**Supplementary material (for online publication)**

**Additional table1 : Micronutrient composition of LMF oil**

| **Composition per unit dose (2.5ml)** | **1-6 months** | **6-12 months** |
| --- | --- | --- |
| Elemental iron | 0.5 mg | 1.5 mg |
| Folate | 40 mcg | 40 mcg |
| Vitamin B12 | 0.3 mcg | 0.3 mcg |
| Vitamin D3 | 400 IU | 400 IU |

Base oil used : sunflower seed oil

**Additional Table2: Tolerability and acceptability of LMF oil in adults and children**

| **Type of study** | **Baseline MIS** | **Post-completion MIS** | **Irritancy assessment** |
| --- | --- | --- | --- |
| 24-hrs irritation patch test amongst adults (n=26) | 1.53 | 0 | Non-irritant |
| 15-days tolerability in adults (n=15) | 0 | 0.033 | Non-irritant |
| 15-days tolerability in children (n=15) | 0.0769 | 0 | Non-irritant |

MIS – Mean irritation score which mainly assesses erythema and dryness at the site of local application, score more than 1 indicates irritation


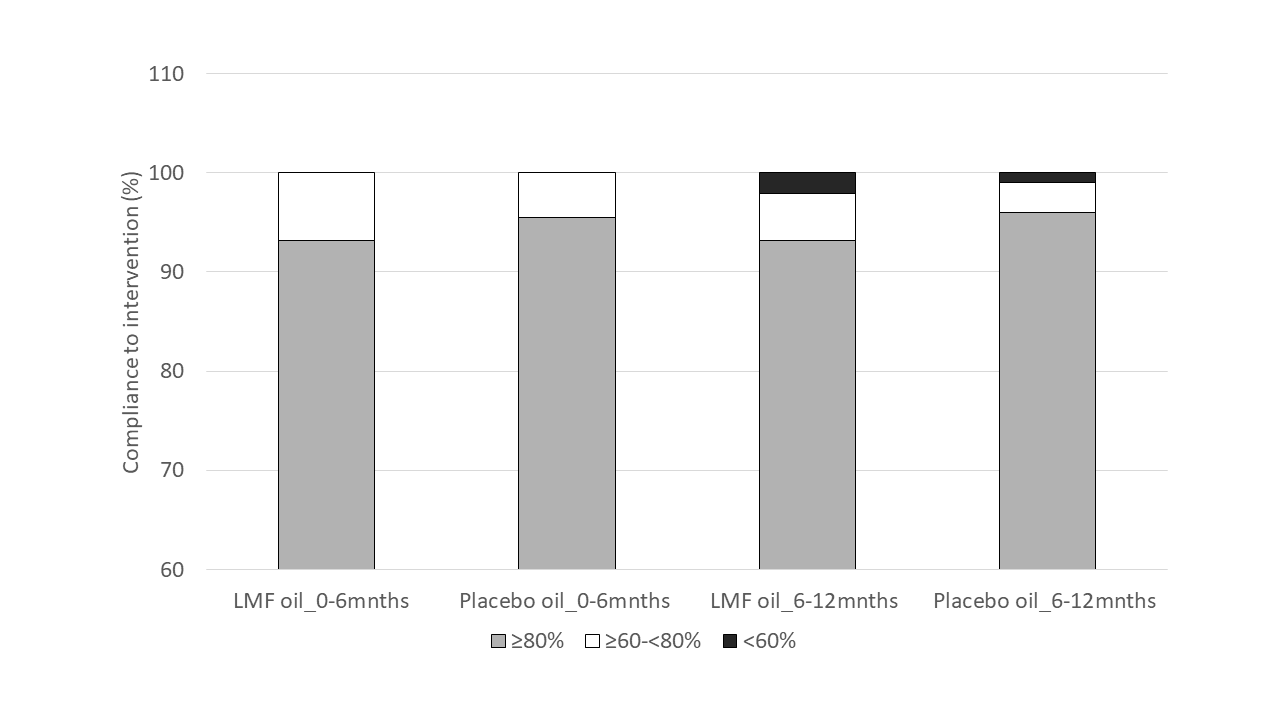
**Additional figure 1: Compliance to the intervention amongst LMF oil and placebo oil group**

**Additional table 3: Anthropometry and Nutritional Parameters at 6 months and 12 months**

|  | **6 months** | | | **12 months** | | |
| --- | --- | --- | --- | --- | --- | --- |
|  | **LMF oil**  **(n=201)** | **Placebo oil**  **(n=204)** | **P value** | **LMF oil**  **(n=190)** | **Placebo oil**  **(n=201)** | **P value** |
| Weight in kg [Mean ± SD] | 7.05 ± 0.83 | 6.80 ± 0.83 | 0.13* | 8.48 ± 1.03 | 8.23 ± 0.97 | 0.08* |
| Height in cm [Mean ± SD] | 65.72 ± 2.65 | 65.50 ± 2.63 | 0.20 | 73.06 ± 2.83 | 72.70 ± 2.76 | 0.10 |
| Head circumference in cm [Mean ± SD] | 41.72 ± 1.49 | 41.44 ± 1.49 | 0.03* | 44.15 ± 1.43 | 43.94 ± 1.49 | 0.08 |
| Exclusive breastfeeding, n(%) | 141(70.15) | 133(65.20) |  | -- | --- |  |
| Age of weaning in months, [Mean ± SD] | 6.08 ± 0.95^1^ | 6.20 ± 0.92^2^  (n=202) |  | -- | ---- |  |

*- Data not distributed normally, non-parametric tests of hypotheses used; ^1^ n=198; ^2^ n =202

**Additional Table 4: Analysis of adverse events**

| **Adverse events, n (%)** | **LMF oil**  **(n=222)** | **Placebo**  **(n=222)** | **P value** |
| --- | --- | --- | --- |
| **No of subjects with at least one AE** | 187 (84.23) | 177(79.72) | 0.20 |
| **Respiratory Diseases/Conditions** |  |  |  |
| Rhinitis | 4 | 6 |  |
| Upper Respiratory Tract Infection | 385 | 312 |  |
| Acute otitis media | 1 | 1 |  |
| Rhinorrhea | 1 | 2 |  |
| Lower Respiratory Tract Infection | 0 | 3 |  |
| Broncho-pneumonitis | 2 | 0 |  |
| **No of subjects with at least one episode of upper respiratory infection** | **168** | **152** | 0.09 |
| **Skin Diseases/Conditions** |  |  |  |
| Pityriasis Alba | 5 | 1 |  |
| Scabies | 1 | 1 |  |
| Skin dryness/Ichthyosis | 2 | 3 |  |
| Other dermatophytosis | 0 | 4 |  |
| Impetigo Contagiosa | 1 | 1 |  |
| Dermatitis | 5 | 3 |  |
| Rash with fever/URTI | 2 | 2 |  |
| Rash on face | 5 | 3 |  |
| Rash all over the body | 13 | 10 |  |
| Rash on abdomen/thighs/neck/back | 4 | 2 |  |
| Genital/Perianal Rash/Diaper rash | 4 | 4 |  |
| Miliria | 2 | 3 |  |
| **Gastro-intestinal Diseases/Conditions** |  |  |  |
| Constipation | 8 | 1 |  |
| Acute Gastroenteritis | 105 | 101 |  |
| Infantile colic | 12 | 11 |  |
| Vomiting | 2 | 1 |  |
| Diarrhea | 3 | 0 |  |
| Anorexia | 1 | 1 |  |
| Stomatitis | 2 | 1 |  |
| **No of subjects with at least one episode of gastroenteritis** | **73** | **64** | **0.35** |
| **Infections/Inflammatory conditions** |  |  |  |
| Chicken Pox | 2 | 1 |  |
| Conjunctivitis | 5 | 0 |  |
| Lymph node swelling | 0 | 1 |  |
| Urethritis | 1 | 0 |  |
| Urinary Tract Infection | 1 | 0 |  |
| Otitis externa | 1 | 1 |  |
| Acute Febrile Illness | 31 | 28 |  |
| Injury (including fracture and dislocation) | 3 | 5 |  |
| Burn | 1 | 0 |  |
| Pain/Local swelling at vaccination | 0 | 2 |  |
| Pain in neck | 1 | 0 |  |
| Dysuria | 1 | 0 |  |
| Otalgia | 2 | 1 |  |
| Swelling behind left ear | 0 | 1 |  |
| Irritability | 11 | 9 |  |
| Insect bite | 0 | 2 |  |
| Redness of both eyes | 0 | 1 |  |
| Epistaxis | 1 | 0 |  |
